# Wide Voltage Swing Potentiostat with Dynamic Analog Ground to Expand Electrochemical Potential Windows in Integrated Microsystems

**DOI:** 10.3390/s24092902

**Published:** 2024-05-01

**Authors:** Ehsan Ashoori, Derek Goderis, Anna Inohara, Andrew J. Mason

**Affiliations:** Department of Electrical and Computer Engineering, Michigan State University, East Lansing, MI 48824, USA; ashoorie@msu.edu (E.A.); goderisd@msu.edu (D.G.); inoharaa@msu.edu (A.I.)

**Keywords:** bidirectional potentiostat, miniaturized CMOS potentiostat, electrochemical instrumentation, wide voltage range potentiostat, integrated microsystem

## Abstract

Electrochemical measurements are vital to a wide range of applications such as air quality monitoring, biological testing, food industry, and more. Integrated circuits have been used to implement miniaturized and low-power electrochemical potentiostats that are suitable for wearable devices. However, employing modern integrated circuit technologies with low supply voltage precludes the utilization of electrochemical reactions that require a higher potential window. In this paper, we present a novel circuit architecture that utilizes dynamic voltage at the working electrode of an electrochemical cell to effectively enhance the supported voltage range compared to traditional designs, increasing the cell voltage range by 46% and 88% for positive and negative cell voltages, respectively. In return, this facilitates a wider range of bias voltages in an electrochemical cell, and, therefore, opens integrated microsystems to a broader class of electrochemical reactions. The circuit was implemented in 180 nm technology and consumes 2.047 mW of power. It supports a bias potential range of 1.1 V to −2.12 V and cell potential range of 2.41 V to −3.11 V that is nearly double the range in conventional designs.

## 1. Introduction

Electrochemical measurements have a wide range of applications in science and technology as well as for the everyday lives of people. For instance, electrochemical tests can be utilized to determine the quality of the food in a supply chain [1,2], assessing human health by analyzing human secretions such as sweat [3,4], detecting precursors for cancer [5], monitoring air quality and detecting toxic gas or particulate matter [6,7] and detecting heavy metals [8,9]. These applications allow people to make informed decisions to enhance their health and quality of life. To best utilize electrochemical methods in many practical applications, it is paramount to deploy these methods in small, low-power, cheap and preferably wearable or point-of-care devices. For instance, rapid, frequent, and cost-effective measurements of health metrics can become widely accessible to individuals. Moreover, by deploying a network of low-cost electrochemical sensors in a densely populated area, rich datasets of air quality with high temporal and spatial resolution can be obtained for improved community health. However, achieving these capabilities requires the development of miniaturized and low-cost electrochemical instruments. To this end, researchers have utilized complementary metal-oxide semiconductor (CMOS) technology to develop small and wearable potentiostats [10,11,12,13], and many advances have been made to develop potentiostats that increase the range of current readout [4,14], decrease the power consumption and size [15,16,17], lower the noise [18] and widen the dynamic range [19], and support the bidirectional current of electrochemical cells [14]. New processes have also been developed for implementing quasi-reference electrodes on the CMOS chip for a fully integrated electrochemical measurement [20].

**Figure 1:**
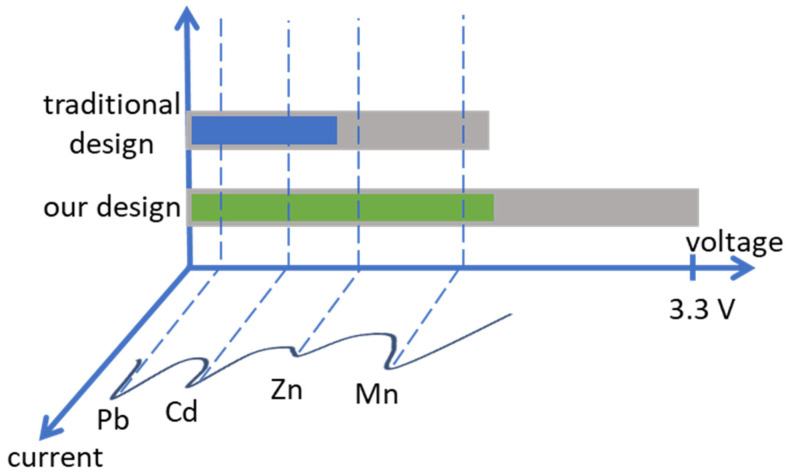
The graph shows voltammetry of different heavy metals that indicates bias potentials for each target element to obtain peak current (adapted from [8]). The blue and green bars show ideal ranges of bias potential that are supported with a traditional CMOS potentiostat and our novel potentiostat, respectively, both with a 3.3 V supply. In this example, the reactions for some elements such as Zn and Mn are not supported by a traditional CMOS potentiostat. Note that the gray bar represents V_CE-swing_, the excess voltage beyond the bias potential required for an electrochemical cell.

Although these advances have enabled miniaturized electrochemical systems, as the modern CMOS technologies scale down in size, the voltage supply has become smaller [21]; for example, while an older 0.5 µm CMOS technology used to support a 5 V supply, newer technologies such as 180 nm support a maximum of 1.8 V for regular transistors or 3.3 V in the case of high-voltage transistors. As a result, many electrochemical reactions cannot be supported by modern integrated potentiostats, as illustrated in Figure 1.

Since a potentiostat needs to support bidirectional current for redox reactions, only half of the supply voltage is available to be used for each direction in an ideal rail-to-rail operation of the potentiostat. For a 3.3 V supply, this means only 1.65 V is available for each reduction or oxidation reaction. Furthermore, as detailed in Section 2, because the counter electrode in a typical three-electrode electrochemical cell must be allowed to swing well beyond the bias potential, only a small portion of this 1.65 V is available to be used as bias potential, as illustrated in Figure 1. However, many electrochemical reactions, for example for detecting heavy metals such as manganese and zinc, require bias potentials of about 1.6 V and 1.2 V, respectively. As shown in Figure 1, these potentials fall outside the window that is supported by conventional CMOS potentiostats with power supplies of 3.3 V or lower. Therefore, conventional CMOS potentiostat designs implemented in newer technologies with lower supply voltages do not support voltammetry for detection of these elements. On the other hand, older CMOS process nodes such as 0.5 µm that support supply voltages greater than 3.3 V are not offered by mainstream foundries as they are considered obsolete [22]. Therefore, it is inevitable to utilize these newer CMOS technologies for electrochemical measurements that come with the added benefits of a smaller feature size, lower power consumption and higher speed. Consequently, overcoming the issue of limited bias potential in CMOS potentiostats implemented in newer process nodes is crucial to accommodate a wide range of electrochemical reactions.

In this paper, we present a novel potentiostat topology that addresses the limited supply voltage in newer CMOS technologies and supports bidirectional current measurement in a wide range of electrochemical reactions. For a given supply voltage, this new topology nearly doubles the voltage range for the electrochemical cell compared to conventional designs. Hence, it enables the detection of a wider range of target elements than any previously reported integrated potentiostat. As desired with most integrated instrumentation circuits, this potentiostat also provides a small form factor and low power consumption for a compact system implementation. The rest of the paper is organized as follows. Section 2 presents an in-depth analysis on the voltage requirements of a three-electrode electrochemical cell as well as the challenges of Conventional CMOS potentiostats. Section 3 presents the methodology and design for enhancing the voltage range of the electrochemical cell. Section 4 presents the results of electrochemical experiments as well as simulation results of the CMOS potentiostat. Finally, Section 5 concludes the paper.

## 2. Manifestation of Electrode Potentials and Challenges for Conventional CMOS Potentiostats

### 2.1. Electrochemical Cell Model and Manifestation of Potentials at Electrodes

**Figure 2:**
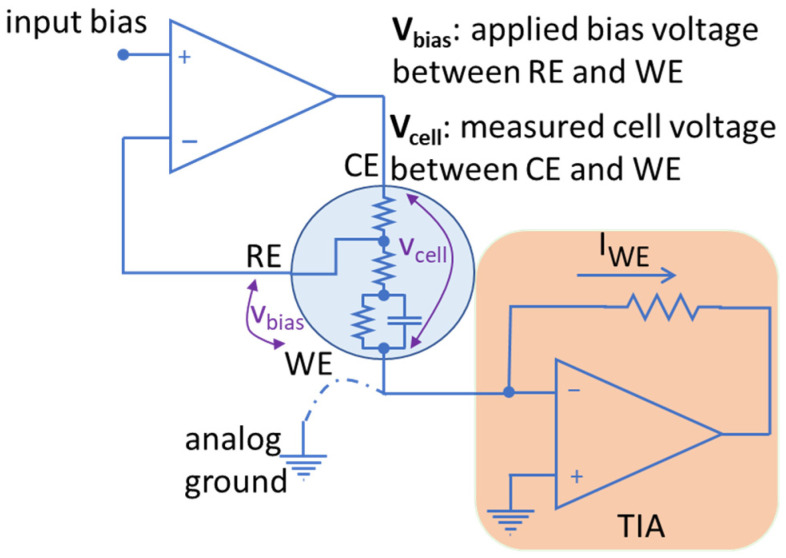
Schematic of a traditional potentiostat with grounded working electrode. The electrochemical cell model is presented at the center of the figure with a circle symbol.

As briefly asserted in Section 1, an important bottleneck in miniaturized CMOS potentiostats is their ability to support a wide bias potential window to extend the range of electrochemical targets that can be measured using CMOS instrumentation. To elaborate on this point, consider the electrochemical cell model shown in the circle at the center of Figure 2. A three-electrode electrochemical cell features a reference electrode (RE), a working electrode (WE) and a counter electrode (CE). The resistance between CE and RE is mainly attributed to the solution resistance. Similarly, the resistance between RE and WE is attributed to the solution resistance in series with a parallel capacitance and resistance that model the double layer capacitance and charge transfer resistance at the WE surface.

In this three-electrode cell, a bias voltage is traditionally applied to the RE with respect to WE. In other words, V_RE-WE_ is applied to the electrochemical cell as shown in Figure 2. In this paper, we will refer to this applied voltage as V_bias_. Note that V_bias_ is sometimes defined as V_WE-RE_ [8], which is thenegative of V_bias_ as defined here. Both definitions are valid as long as one remains consistent. Therefore, throughout this paper, we define:

V_bias_ = V_RE-WE_ = V_RE_ − V_WE_ (1)

This definition facilitates a clearer discussion about the integrated CMOS potentiostat. While the V_bias_ is externally applied between RE and WE, the potential on CE can and will swing beyond V_bias_ in order to establish a desired electrochemical reaction. Let us define this CE swing voltage as:

V_CE-swing_ = V_CE-RE_ = V_CE_ − V_RE_ (2)

This V_CE-swing_ depends on several factors such as electrolyte concentration and the geometry and material of electrodes, and it can be as large as V_bias_, which extends the maximum potential the potentiostat must support to beyond two times V_bias_. Finally, let us define the full cell potential, V_cell_ such that:

V_cell_ = V_CE-WE_ = V_CE_ − V_WE_ = V_CE-swing_ + V_bias_ (3)

**Figure 3:**
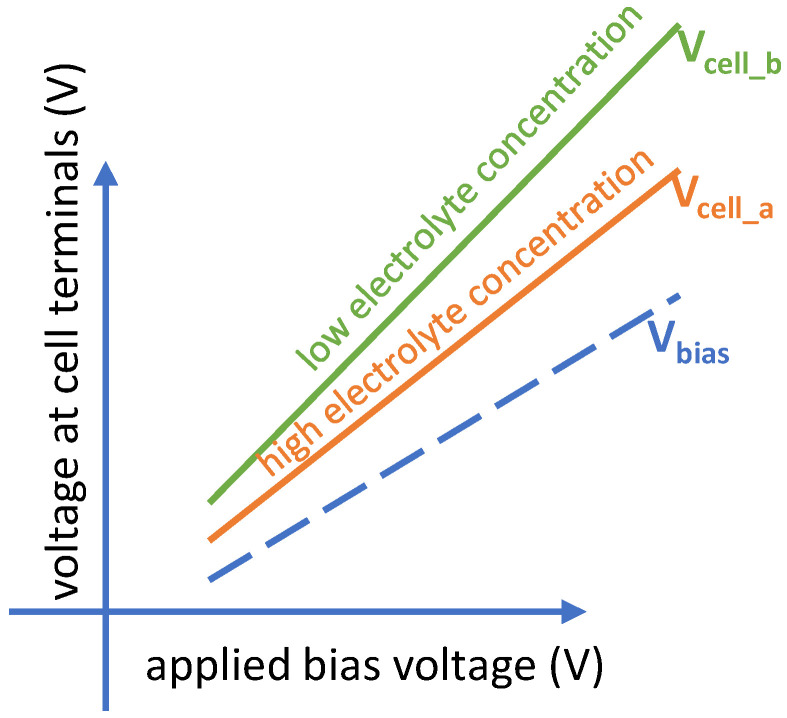
Conceptual representation of V_cell_ and V_bias_. V_RE-WE_ (V_bias_) is always equivalent to the V_bias_ voltage applied to the electrochemical cell. V_CE-WE_ (V_cell_), however, is more than V_bias_ and further increases if electrolyte concentration decreases.

Based on our extensive experience with integrated electrochemical platforms, we expect voltages at the cell electrodes to generally manifest similar to the graph in Figure 3. The absolute value of the cell potential is always more than that of the bias potential due to the existence of CE-RE resistance. Moreover, by lowering the electrolyte concentration, the CE-RE potential difference further increases due to the increase in the CE-RE resistance. Therefore, for a potentiostat with a limited voltage supply, the voltage swing on CE is the limiting factor.

### 2.2. Challenges of Conventional CMOS Potentiostats

A conventional CMOS potentiostat is shown in Figure 2. An operational amplifier is used to apply a bias voltage to an electrochemical cell. The current generated in the electrochemical cell is usually read using a transimpedance amplifier (TIA) as shown in the bottom right of Figure 2. The WE of the electrochemical cell in this design is tied to analog ground which is usually set to V_supply_/2. This allows the potentiostat to support bidirectional current measurement and hence supports both reduction and oxidation reactions. For instance, in the old 0.5 µm CMOS technology with a 5 V supply, in an ideal rail-to-rail operation of the circuit, the analog ground is set to 2.5 V. Therefore, the available voltage for |V_cell_| is 2.5 V in either direction (negative or positive). Basically, the bottom half of the supply range (0 V to 2.5 V) is used to support negative V_cell_ (remember V_cell_ = V_CE_ − V_WE_) and the top half (2.5 V to 5 V) is used to support positive V_cell_. Only a portion of this 2.5 V in either direction can be assigned to V_bias_ because always V_bias_ < V_cell_ (the exact ratio of V_bias_ to V_cell_ depends on the cell condition such as electrolyte concentration). This covers a relatively wide range of electrochemical experiments [23]. However, 0.5 µm CMOS process node is not offered by major foundries anymore [22]. On the other hand, the supply voltage in newer CMOS technologies is drastically reduced compared to the older technologies. For example, going from 0.5 µm CMOS to a newer 180 nm CMOS, the supply voltage drops from 5 V to 1.8 V (or 3.3 V in case of high-voltage transistors). This reduction in supply severely restricts the range of electrochemical experiments that can be conducted using a conventional CMOS potentiostat. In other words, this reduced supply voltage is not sufficient to support V_bias_ and V_cell_ in an electrochemical cell. In this case, for an ideal rail-to-rail operation of a CMOS potentiostat with 3.3 V supply, only V_supply_/2 = 1.65 V is available for the electrochemical cell in either direction (negative or positive). Therefore, the absolute value for the maximum |V_cell_| = |V_CE_ − V_WE_| in this case is 1.65 V (i.e., ‘V_supply_ − analog ground’ or ‘analog ground − gnd’). |V_bias_| = |V_RE_ − V_WE_| in this case will be much lower than |V_cell_| as described in the previous section. The results of our experiments suggest V_bias_ ≈ 0.5 V_cell_ as presented in Section 4, but the exact ratio depends on the characteristics of the electrochemical cell. Consequently, only around 0.9 V is available as V_bias_ in this example with a conventional potentiostat. As shown in Figure 1, many of the electrochemical reactions happen in bias voltages outside this potential window [8,23,24] and hence are not supported by conventional methods. In this work, we present a novel circuit architecture for CMOS potentiostats that widens the supported windows for V_bias_ and V_cell_ to facilitate a wide range of electrochemical reactions.

## 3. Design Methodology for CMOS Potentiostats to Support High Voltage Requirements

**Figure 4:**
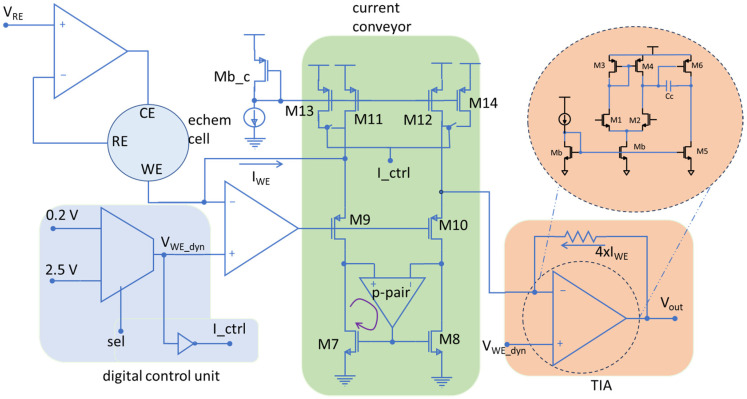
Schematic of the implemented potentiostat. The current conveyor in the middle of the schematic is employed to reverse the direction of current to support bidirectional current measurement while allowing WE to switch between supply rails.

To solve the problem of a limited potential window, a novel architecture is introduced in this work to enable a wider range of electrochemical experiments using cutting-edge CMOS technologies. The first step to widen the voltage swing is to allow the voltage on the WE to switch between high and low supply rails, instead of being tied to analog ground. This will allow V_bias_ to have a voltage swing of the full supply range in an ideal rail-to-rail operation of the circuit. The limitation, however, arises in reading the current. Traditionally, a TIA is used to read the current, whose reference point is tied to analog ground together with the WE as shown in Figure 2. By employing the proposed method, the reference point of the TIA should switch between high and low supply rails along with WE. However, this does not allow reading current in the original direction as it will push the output of the TIA beyond 3.3 V (the supply voltage) or less than 0 V (ground) which is not possible. Therefore, a current conveyor was employed to reverse the direction of the current and thus enable the TIA to read the current properly. The schematic of the current conveyor is seen in the middle of Figure 4.

### 3.1. Current Conveyor

The current conveyor in this work was designed with the objective of enabling wide output voltage swing. In a typical current conveyor, a cascode current mirror is used to ensure the accuracy of the copying current from the left leg to the right leg. However, to maximize the voltage swing at the output, a single transistor was used in the current mirror to reduce the overhead voltage required for the circuitry and hence maximize the voltage swing for electrochemical reactions. These single transistors (M7 and M8) are shown in the bottom center of Figure 4. However, using single transistors results in mismatch between mirrored currents if the transistor’s drain voltages do not follow each other. To ensure matching of the current in both legs of the current mirror, an op amp was employed to match the drain voltages of the transistors. The input pair of this op amp was constructed of PMOS transistors to ensure that the op amp remains in saturation mode even with low voltages at its input terminals. This op amp is placed in both positive and negative feedback loops. As the impedance at the left leg of the current conveyor is higher than that of the right leg, the positive terminal of the op amp is connected to the left side to form a negative feedback loop (as shown with the purple curved arrow in Figure 4) that is stronger than the positive feedback loop. Note that since M7 adds 180 degrees to the phase, the drain of M7 is tied to the positive terminal of the op amp to ensure a strong negative feedback loop. This guarantees the stability of the circuit, and it ensures the drain voltages of the two transistors match and the current is accurately mirrored.

**Figure 5:**
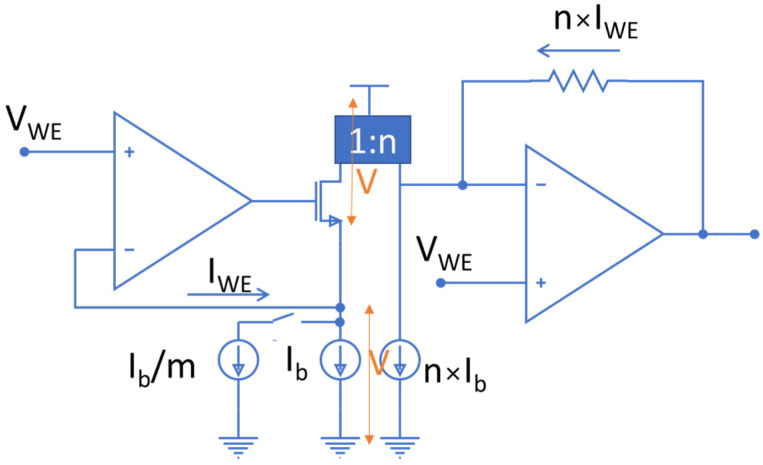
Initial design of the current conveyor with NMOS transistors. This design suffered from the limited voltage range available for the electrochemical cell that was mainly caused by the body effect of the NMOS transistor.

To maximize the voltage range for electrochemical cells, the transistors at the output of the op amp in the current conveyor (M9 and M10) should be carefully designed. The first option considered was the NMOS-based design shown in Figure 5. However, the high threshold and overdrive voltages of the NMOS transistor at the output of the op amp was found to limit the available voltage for electrochemical reactions. This higher threshold voltage was due to the body effect of the NMOS transistor. To reduce the overdrive voltage that limits output swing, it was noted that a design based on PMOS transistors in isolated n-wells would eliminate the body effect and hence decrease the threshold and overdrive voltages of the transistors, given our design utilizes an n-well CMOS technology. Therefore, to decrease overdrive voltage and increase the range of voltage available for electrochemical reactions, the PMOS transistors with isolated wells were employed in the final design, as depicted in Figure 4. In addition, a PMOS transistor was added to the middle of the second leg of the current conveyor to balance the current in both legs.

### 3.2. Digital Control Unit

A digital control unit was employed to dynamically change the reference voltage that is applied to WE (bottom left of Figure 4) and the positive terminal of the TIA (right side of Figure 4). Also, a digital signal (I_ctrl) was created from the WE voltage, and this signal was used to control the current sources employed in the current conveyor, through the switches depicted in the top center of Figure 4. This control of the bias current is crucial to properly bias the current conveyor according to the voltage that is applied to the WE.

## 4. Results

### 4.1. Test Setup and Electrochemical Experiments

To assess the behavior of electrode voltages for varying electrochemical model parameters, chronoamperometry experiments were conducted using different electrolyte concentrations. Electrodes in these experiments were built in-house using standard microfabrication techniques, including photolithography and thermal evaporation. Interdigitated electrodes were made by depositing 10 nm of titanium and 100 nm of gold on a silicon wafer containing a thin silicon dioxide layer. The titanium was used as an adhesion layer between the gold and the oxide substrate. For the electrolyte, phosphate buffer (PB) solution was used in low concentrations to increase the lifetime of thin-film gold electrodes. Phosphate buffer saline (PBS) solution was avoided because the chlorine (Cl) molecules released from saline were observed to dissolve the gold electrodes in previous experiments.

**Figure 6:**
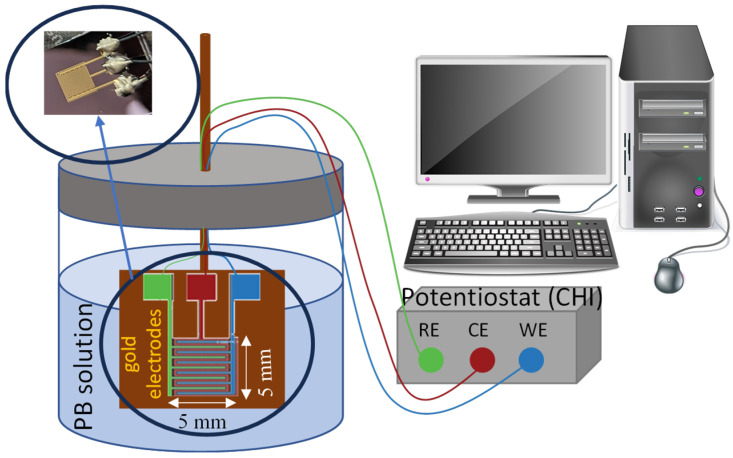
Schematic of the experimental setup for concentration comparison using interdigitated electrodes and CHI potentiostat. A photo of the fabricated electrodes is shown in-set. The size of electrode is 5 mm × 5 mm.

**Figure 7:**
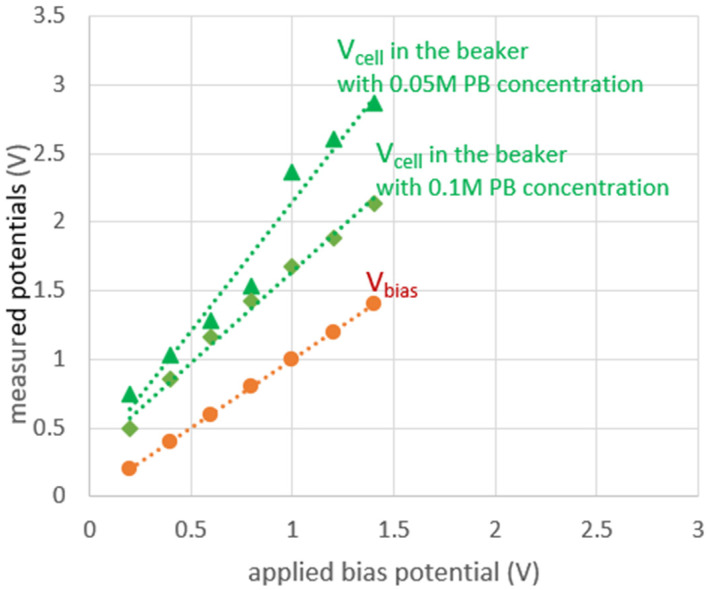
Measured CE and RE voltages w.r.t. the WE voltage (i.e., V_cell_ and V_bias_). V_cell_ is always higher than V_bias_ and this voltage difference increases as electrolyte concentration decreases.

Experiments with our custom interdigitated gold electrodes were performed in a beaker using 0.05 M and 0.1 M PB solutions. A commercial electrochemistry instrument (CHI 760E) was used for chronoamperometry measurements. An illustration of the test setup and electrodes are shown in Figure 6, where a photo of the fabricated interdigitated electrode is provided as an inset. The experimental measurements shown in Figure 7 confirm the initial expectation that, while V_bias_ (V_RE-WE_) stays at the applied bias potential, V_cell_ (V_CE-WE_) is always greater than the applied bias potential. Notice also from Figure 7 that the CE potential further increases when the electrolyte concentration is decreased. For instance, for a V_bias_ of 1.4 V, a V_cell_ of 2.2 V and 2.9 V were measured for high and low electrolyte concentrations, respectively. This validates the importance of expanding the potential window that a potentiostat could support.

### 4.2. Electrochemical Cell Model

**Figure 8:**
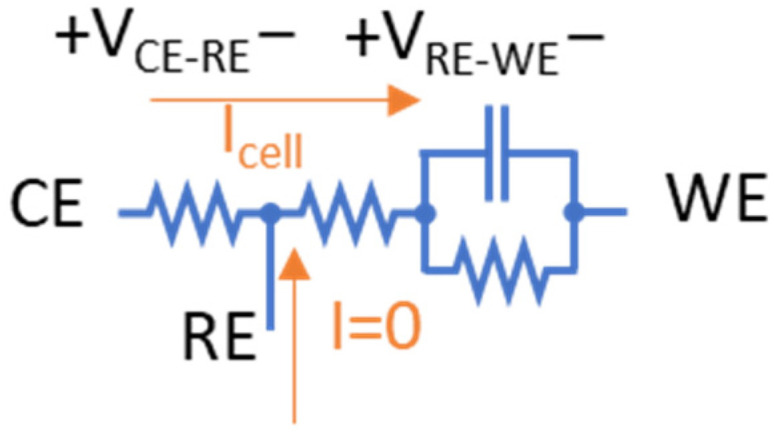
The electrochemical cell characterization for simulation of the potentiostat. The characterization was performed using the measured voltage and current using the CHI 760E instrument.

As described in Section 2.1. the parallel capacitance and resistance in Figure 8 model the double layer capacitance and charge transfer resistance at the WE surface. For all simulations of the novel wide-swing potentiostat, a typical value of 2.6 µF was chosen as the model capacitance and a value of 64 kΩ was used to model the charge transfer resistance based on the data presented in [25]. The solution resistance values of the electrochemical cell were empirically modeled from the experiments described in Section 4. 1 as follows: the measured steady state chronoamperometry current for a given bias voltage was used to calculate the RE-WE resistance, and the measured cell voltage for each chronoamperometry current was used to determine CE-RE resistance. For these calculations, measurements were performed at V_bias_ = 1 V where V_cell_ was measured as 2.44 V. Then, the RE-WE resistance was calculated as 10.2 MΩ and the CE-RE resistance was calculated as 14.7 MΩ. This calculation is based on the fact that the RE in a three-electrode electrochemical cell does not draw any current [26]. Therefore, the resistances in the electrochemical cell can be considered to be in series and the voltage drop on the resistances can be calculated by Kirchhoff’s circuit laws. The voltage and current distribution on the model electrochemical cell are depicted in Figure 8. This gives a reasonable approximation of resistance values that were used in simulations.

### 4.3. Simulation Results for the CMOS Potentiostat

Using the transistor sizes listed in Table 1, the new potentiostat design from Figure 4 was simulated in Cadence along with the electrochemical cell model described above.

**Figure 9:**
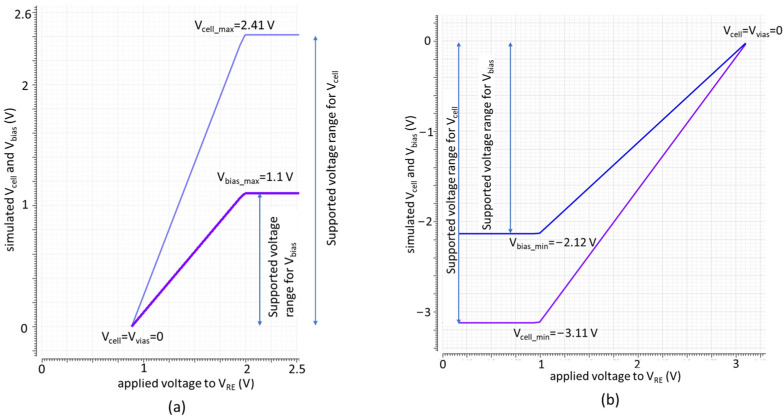
Simulated voltage range for V_bias_ and V_cell_. The graphs show the enhanced supported voltage range for (**a**) positive and (**b**) negative V_bias_ and V_cell_.

The simulation results are shown in Figure 9. Figure 9a demonstrates the voltage support for positive V_bias_ and V_cell_. In this case, the digital control unit fixes the WE voltage at 0.88 V. By sweeping RE voltage from 0.88 V toward 3.3 V, V_bias_ increases until it saturates at V_bias_max_ = 1.1 V. Consequently, V_cell_ also increases until it saturates at V_cell_max_ = 2.41 V. Likewise, Figure 9b explains voltage support for negative V_bias_ and V_cell_. In this case, the WE voltage is fixed at 3.2 V and the RE voltage is swept from 3.2 V toward 0 V. As seen in Figure 9b, a V_bias_ of −2.12 V to 0 V and a V_cell_ of −3.11 V to 0 V are supported. This demonstrates that the new potentiostat enhances the potential window for oxidation and reduction measurements by supporting a maximum V_cell_ of 2.41 V and −3.11 V in positive and negative directions, respectively. In comparison, a conventional potentiostat, even with an ideal rail-to-rail operation at a 3.3 V supply supports a maximum V_cell_ of only ±1.65 V. Therefore, our new potentiostat architecture achieves a 46% and 88% increase in the voltage range of V_cell_ for positive and negative voltages, respectively.

**Figure 10:**
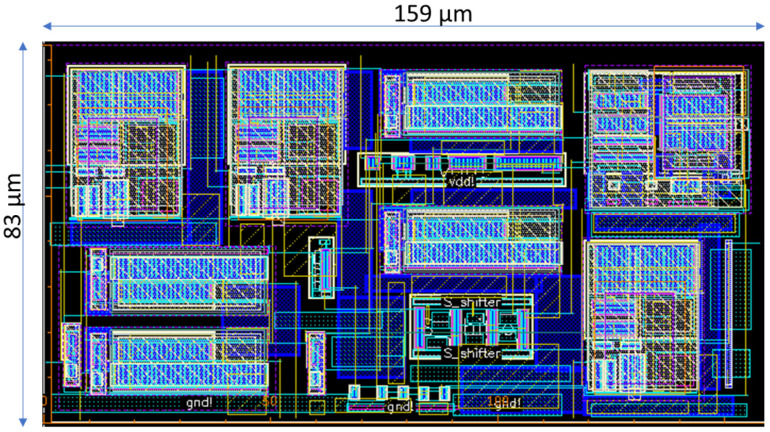
Layout of the entire potentiostat designed in 0.18 µm CMOS technology. The total dimension is 159 µm × 83 µm.

The design operates from a 3.3 V supply and consumes only 2.047 mW of power. Figure 10 shows the layout of the new potentiostat designed in 180 nm CMOS technology, which occupies only 0.013 mm^2^. Table 2 highlights the design and performance characteristics of the op amp designed for and employed in the potentiostat, and Table 3 illustrates the characteristics of the whole potentiostat including the current conveyor, the digital control unit and the TIA. The 10% to 90% charge and discharge time of a typical 2.6 µF capacitor within the model electrochemical cell was less than 1 µs. Considering the reaction times of multiple seconds in a typical chronoamperometry experiment, the charge and discharge times are negligible and the potentiostat meets the speed requirements.

## 5. Conclusions

Integrated circuits have been used to implement low-power potentiostats with small form factor that can be used for wearable devices and play a key role in many applications such as air quality monitoring and health assessments. However, integrated potentiostats support a limited cell voltage range, V_cell_, that fails to accommodate many electrochemical reactions of interest. To resolve this challenge, we introduced a novel integrated potentiostat topology that was verified to support V_cell_ range between 2.41 V and −3.11 V (with 3.3 V supply). This increases the maximum supported V_cell_ by 46% and 88% for positive and negative voltages, respectively, compared to a traditional potentiostat design. This dramatic improvement in potential window permits the measurement of a much wider range of electrochemical targets, expanding applications for portable sensing systems. The circuit was implemented in CMOS 180 nm technology and consumes only 2.047 mW of power. For a given electrochemical cell model, the maximum charge and discharge time was found to be under 1 µs, easily meeting the speed requirements for most electrochemical experiments. The greatly expanded potential window of this new potentiostat, along with its low power consumption and high slew rate, make this design well-suited for many current and future wearable electrochemical sensing platforms.

## Figures and Tables

**Table 1:** Transistor sizing of the CMOS potentiostat.

Device	W/L	Fingers	Device	W/L	Fingers
M1,2	4 u/500 n	1	M8	4 × 2 u/350 n	1
M3,4	8.5 u/500 n	2	M9	8 u/300 n	1
M5	7.5 u/500 n	4	M10	4 × 8 u/300 n	1
M6	10 u/500 n	20	M11	2 u/300 n	2
Mb	6 u/500 n	1	M12	2 u/300 n	8
Mb_c	2 u/300 n	2	M13	2 u/300 n	4
M7	2 u/350 n	1	M14	2 u/300 n	16

**Table 2:** Characteristics of the op amp.

Supply	Technology	Area	Power	Bandwidth	Slew Rate
3.3 V	CMOS 180 nm	964 µm^2^	435 µW	32.52 MHz	28.33 V/µs

**Table 3:** Electrical characteristics of the potentiostat.

Supply	Area	Max Power	Max Cell Voltage Support	Load Capacitance
3.3 V	0.0132 mm^2^	2.047 mW	3 V	2.6 µF

## Data Availability

The original contributions presented in the study are included in the article, further inquiries can be directed to the corresponding author.
